# Multi-Party Quantum Byzantine Agreement without Entanglement

**DOI:** 10.3390/e22101152

**Published:** 2020-10-14

**Authors:** Xin Sun, Piotr Kulicki, Mirek Sopek

**Affiliations:** 1 Department of the Foundations of Computer Science, John Paul II Catholic University of Lublin, 20-950 Lublin, Poland; xin.sun.logic@gmail.com; 2 MakoLab SA, 91-062 Lodz, Poland; sopek@makolab.com

**Keywords:** Byzantine agreement, quantum communication, distributed computing

## Abstract

In this paper, we propose a protocol of quantum communication to achieve Byzantine agreement among multiple parties. Our protocol’s striking feature compared to the existing protocols is that we do not use entanglement to achieve the agreement. The role played by entangled states in other protocols is replaced in our protocol by a group of semi-honest list distributors. Such a replacement makes the implementation of our protocol more feasible. Moreover, our protocol is efficient in the sense that it achieves agreement in only three rounds which is a significant improvement with respect to the alternative agreement protocol not using entanglement. In the first round, a list of numbers that satisfies some special properties is distributed to every participant by list distributors via quantum secure communication. Then, in the second and third rounds, those participants exchange some information to reach an agreement.

## 1. Introduction

A fundamental problem in distributed computing is how to reach an agreement in the presence of faulty processes. For example, a database can be replicated on several computers, which ensures access to the database even if some of the computers are not functional. For the consistency of data, all computers must preserve the same contents. To achieve this goal, a protocol that ensures that all computers adopt the same update of the database is needed. This problem is intuitively formulated as the Byzantine generals problem:

“Three generals of the Byzantine army want to decide upon a common plan of action: either to attack (0) or to retreat (1). They can only communicate in pairs by sending messages. One of the generals, the commanding general, must decide on a plan of action and communicate it to the other generals. However, one of the generals might be a traitor, trying to keep the loyal generals from agreeing on a plan. How to find a way in which all loyal generals follow the same plan?”

If the generals communicate with each other only by pairwise classical channels, the Byzantine generals problem is provably unsolvable [1,2]. Even if pairwise quantum channels are used, it will not help to solve the problem [3]. However, a variation of the Byzantine agreement problem, called detectable Byzantine agreement (DBA), can be solved using quantum resources. A DBA protocol ensures that either all loyal generals agree upon a common plan or all abort. In addition, if all generals are loyal, then they agree upon a common plan.

In 2001, Fitzi et al. [4] presented a DBA protocol for three parties using pairwise quantum channels and entangled qutrits. Cabello [5] proposed a three-party DBA protocol based on a four-qubit singlet state. Iblisdir and Gisin [6] developed an improvement of the protocol of Fitzi et al. [4] by showing that the DBA problem can be solved by using two quantum key distribution channels and three classical authenticated channels. Gaertner et al. [7] introduced a new DBA protocol based on four-qubit entangled state. An experimental implementation of the protocol was also presented by Gaertner et al. [7]. A device-independent quantum scheme for the Byzantine generals problem was provided by Rahaman et al. [8].

All the aforementioned DBA protocols only consider the situation of three parties. In actual distributed computing or in blockchains [9,10], the number of parties involved is significantly larger than three. Ben-Or and Hassidim [11], Tavakoli et al. [12] and Luo et al. [13] developed DBA protocols for multiple parties based on high-dimensional entangled states. These states are difficult to realize by the current technology. In this paper, we develop a new DBA protocol for multiple parties. The crucial feature of our protocol compared to the existing ones is that no entanglement is used in an essential way. The role played by entangled states in other protocols is replaced by a group of semi-honest list distributors in our protocol. Such a replacement makes our protocol easier to implement. The quantum technology that we use is Quantum Key Distribution (QKD) [14], which is a relatively mature technology and an active topic of research and has recently attracted the industry’s interest. It is important to note that, in some QKD protocols (e.g., [15]), the phenomenon of entanglement is used directly on the physical level, and in others entanglement is used to assist and improve standard QKD protocols such as BB84 [16,17]. However, our quantum Byzantine Agreement protocol uses QKD on the data transmission level, so the entanglement usage on the physical level is not relevant on the essential level of our protocol.

To the best of our knowledge, the only existing work on DBA without using entanglement was presented by Fitzi et al. [18]. While the protocol of Fitzi et al. [18] requires f+5 rounds to reach an agreement, where *f* is the number of faulty parties, our protocol is more efficient in the sense that agreement can be achieved in three rounds.

The structure of this paper is as follows. In Section 2, we introduce our protocol. Then, in Section 3, we analyze the properties of our protocol. We conclude the paper with future work in Section 4.

## 2. Quantum Byzantine Agreement without Entanglement

Let us begin with formal definitions of Byzantine agreement.

**Definition** **1.** *[Byzantine agreement (BA) protocol [4]] A protocol among n parties such that one distinct party S (the sender) holds an input value $x_{s}\in D$ (for some finite domain D) and all other parties (the receivers) eventually decide on an output value in D is said to achieve Byzantine agreement if the protocol guarantees that all honest parties decide on the same output value y∈D and that $y=x_{s}$ whenever the sender is honest.*

**Definition** **2.** *[Detectable Byzantine agreement (DBA) protocol [4]] A protocol among n parties such that one sender S holds an input value $x_{s}\in D$ and all other receivers eventually decide on an output value in D is said to achieve detectable Byzantine agreement if the protocol guarantees the following:*

 *1.*  *Agreement: Either all honest parties abort the protocol or all honest parties decide on the same output value y∈D.*
 *2.*  *Validity: If all parties are honest, then they decide on the same output value $y=x_{s}$.*

We assume the parties are pairwise connected by a classical channel and a quantum channel. Both channels are error-free and synchronous. Being synchronous means that parties share a discrete global clock that starts out at time 0 and advances by increments of one. Communication proceeds in a sequence of rounds, with round *k* taking place between time k−1 and time *k*. In each round, every party first sends the messages it needs to send to other parties, and then it receives the messages that were sent to it by other parties in the same round. The classical channels are further assumed to be authenticated, which means that every party can always send classical messages directly to every other party. The adversary can neither prevent those messages from being sent nor introduce new messages on the channel. The quantum channels, however, are insecure in the sense that quantum messages may be tampered by the adversary. Those assumptions ensures that parties can pairwise establish secret keys by quantum key distribution. Indeed, we assume that secret keys with sufficient length are already established before our protocol starts. These assumptions also ensure the parties to create unconditionally secure signatures [19,20] for the communication of classical information. Thus, we assume that all classical messages in our protocol are signed by an unconditionally secure signature scheme.

Now, we introduce our protocol. It consists of three rounds. The aim of the first round is to distribute correlated lists of numbers among the parties involved in the protocol. We call such a list *reference list* since the parties refer to that lists to check whether the information they receive is trustworthy. Then, in the second and third rounds, parties use the reference lists to achieve consensus. We assume the existence of semi-honest parties to handle the task of reference list distribution. This assumption is similar to the one made by Luo et al. [13]. For a party to be semi-honest means that the party acts according to the description of the protocol, but may disclose information with a certain probability *p*, 0<p<1.

### 2.1. Round 1: List Distribution

Let $\left\{P_{1},\ldots ,P_{n},P_{n+1},\ldots ,P_{n+d}\right\}$ be a set of parties. Let further $P_{1}$ be the sender of the DBA protocol, $P_{2},\ldots ,P_{n}$ be receivers and $P_{n+1},\ldots ,P_{n+d}$ be list distributors. To distinguish the sender and the receivers from the distributors, we also call the former two *participants*. We assume that $P_{n+1},\ldots ,P_{n+d}$ are semi-honest. For every party $P_{i}\in \left\{P_{n+1},\ldots ,P_{n+d}\right\}$, the task of $P_{i}$ is to use the technique of quantum secure communication (communicate with the encryption/decryption keys distributed by quantum key distribution) to send a list of numbers $L_{k}^{i}$ (a reference list) to each $P_{k}\in \left\{P_{1},\ldots ,P_{n}\right\}$ such that the following is satisfied:

1. For all k∈{1,…,n}, $|L_{k}^{i}|=m$ for some integer *m* which is a multiple of 6.
2. $L_{1}^{i}\in {\left\{0,1,2\right\}}^{m}$. $\frac{m}{3}$ numbers on $L_{1}^{i}$ are 0. $\frac{m}{3}$ numbers on $L_{1}^{i}$ are 1. $\frac{m}{3}$ numbers on $L_{1}^{i}$ are 2.
3. For all k∈{2,…,n}, $L_{k}^{i}\in {\left\{0,1\right\}}^{m}$.
4. For all j∈{1,…,m}, if $L_{1}^{i}\left[j\right]=0$, then $L_{2}^{i}\left[j\right]=\ldots =L_{n}^{i}\left[j\right]=0$.
5. For all j∈{1,…,m}, if $L_{1}^{i}\left[j\right]=1$, then $L_{2}^{i}\left[j\right]=\ldots =L_{n}^{i}\left[j\right]=1$.
6. For all j∈{1,…,m}, if $L_{1}^{i}\left[j\right]=2$, then for all k∈{2,…,n} the probability that $L_{k}^{i}\left[j\right]=0$ and that $L_{k}^{i}\left[j\right]=1$ are equal (i.e., the numbers of occurences of 0 and 1 are equal in the list).
7. For all j,k∈{1,…,m}, $L_{j}^{i}=L_{k}^{i}$.

Distributors create their lists independently; thus, for different *i* and *j*, the lists $L_{1}^{i}$ and $L_{1}^{j}$ may be different (indeed, the probability that they are the same is quite small).

After the lists are distributed, $P_{1},\ldots ,P_{n}$ use sequential composition to form a longer list to be used in the next stage: $L_{1}=L_{1}^{n+1}\ldots L_{1}^{n+d},\ldots ,L_{n}=L_{n}^{n+1}\ldots L_{n}^{n+d}$.

Obviously, $L_{2}=L_{3}=\ldots =L_{n}$. We call the longer lists *combined reference lists*. Notice that every distributor contributes $\frac{1}{d}$ to the combined reference lists.

### 2.2. Rounds 2 and 3: Reaching Agreement

Now, the parties $P_{1},\ldots ,P_{n}$ run the following steps to reach an agreement:

Round 2 $P_{1}$ sends a binary number $b_{1,k}$ to all $P_{k}$, k∈{2,…,n}. Together with $b_{1,k}$, $P_{1}$ sends to $P_{k}$ the list of numbers $ID_{1,k}$, which indicate all positions of $b_{1,k}$ on the list $L_{1}$. The length of $ID_{1,k}$ is to be $\frac{md}{3}$, where md is the length of $L_{1}$. $P_{1}$ uses $b_{1,k}$ as the final value it outputs.
Round 3 $P_{k}$ checks the obtained message $\left(b_{1,k},ID_{1,k}\right)$ against his own reference list $L_{k}$. If the analysis of $P_{k}$ shows that $\left(b_{1,k},ID_{1,k}\right)$ is consistent with $L_{k}$, then he sets $\left(b_{k,j},ID_{k,j}\right):=\left(b_{1,k},ID_{1,k}\right)$ and sends $\left(b_{k,j},ID_{k,j}\right)$ to all other receivers $P_{j},j\in \left\{2,\ldots ,n\right\}$. Here, $\left(b_{1,k},ID_{1,k}\right)$ is consistent with $L_{k}$ means that for all index $x\in ID_{1,k}$, $L_{k}\left[x\right]=b_{1,k}$. However, if $\left(b_{1,k},ID_{1,k}\right)$ is not consistent with $L_{k}$, then $P_{k}$ immediately ascertains that $P_{1}$ is dishonest and sends to other receivers $P_{j},j\in \left\{2,\ldots ,n\right\}$ message: ⊥, meaning: “I have received an inconsistent message”. To acknowledge the fact that every receiver knows his own output, we formally assume that each of them receives a message from himself.
Time 3 After all messages have been exchanged between the receivers, every $P_{k}$ analyzes the data received from $P_{2},\ldots ,P_{n}$ and acts according to the following criteria:
  (a) If there is a set of receivers *H* with |H|≥2 such that, for all j∈H, $\left(b_{j,k},ID_{j,k}\right)$ is consistent with $L_{k}$, and for some i,j∈H, $b_{i,k}\neq b_{j,k}$, then $P_{k}$ sets his output value to be ⊥.
  (b) If there is a set of receivers *H* with |H|≥2 such that for all j∈H, $\left(b_{j,k},ID_{j,k}\right)$ is consistent with $L_{k}$ and all $b_{j,k}$ are the same, and for all i∉H, $\left(b_{i,k},ID_{i,k}\right)$ is not consistent with $L_{k}$, then $P_{k}$ sets his output value to be $b_{j,k}$.
  (c) If there is a set of receivers *H* with |H|≥2 such that for all j∈H, $\left(b_{j,k},ID_{j,k}\right)$ is consistent with $L_{k}$ and all $b_{j,k}$ are the same, and for all i∉H, the message sent by $P_{i}$ is ⊥, then $P_{k}$ sets his output value to be $b_{j,k}$.
  (d) In all other cases, $P_{k}$ sets his value to be ⊥.

Criteria (a)–(d) are crucial for our protocol. Let us now briefly explain the rationale behind them. In a nutshell, the most important factor here is the following claims:

**Theorem** **1.** *For all k,j∈{2,…,n}, $P_{k}$ believes that $P_{j}$ is honest whenever $\left(b_{j,k},ID_{j,k}\right)$ is consistent with $L_{k}$.*

**Proof.**  We prove the theorem by showing that if $P_{j}$ is dishonest then the probability that $P_{j}$ sets $\left(b_{j,k},ID_{j,k}\right)$ to be consistent with $L_{k}$ is extremely small, when $b_{j,k}\neq b_{1,j}$.

Suppose $P_{j}$ is dishonest and $b_{j,k}\neq b_{1,j}$. Now, $P_{j}$ wants to construct the message $\left(b_{j,k},ID_{j,k}\right)$ and send to $P_{k}$ such that $\left(b_{j,k},ID_{j,k}\right)$ is consistent with $L_{k}$. Note that, in $L_{j}=L_{k}$, there are $\frac{md}{2}$ positions on which $b_{j,k}$ appears. However, on $L_{1}$, there are only $\frac{md}{3}$ positions on which $b_{j,k}$ appears. We say that a position *x* is a *discord* position iff $L_{1}\left[x\right]=2$. If $P_{j}$ selects a discord position *x* and puts it into $ID_{j,k}$, then with probability $\frac{1}{3}$ it will be that $L_{k}\left[x\right]\neq b_{j,k}$. To ensure that $\left(b_{j,k},ID_{j,k}\right)$ is consistent with $L_{k}$, $P_{j}$ has to make a correct choice on all indexes. Therefore, the probability of making a correct choice on all indexes is ${\left(\frac{2}{3}\right)}^{\frac{md}{3}}$, which is extremely small when md is relatively large. Therefore, if it is the case that $\left(b_{j,k},ID_{j,k}\right)$ is consistent with $L_{k}$, then $P_{k}$ can conclude that $P_{j}$ is honest. □

**Theorem** **2.** *For all k,j∈{2,…,n}, if $P_{j}$ is honest and he sends $\left(b_{j,k},ID_{j,k}\right)$ to $P_{k}$, then $\left(b_{j,k},ID_{j,k}\right)$ is consistent with $L_{k}$.*

**Proof.**  If $P_{j}$ is honest and he sends $\left(b_{j,k},ID_{j,k}\right)$ to $P_{k}$, then $\left(b_{j,k},ID_{j,k}\right)=\left(b_{1,j},ID_{1,j}\right)$ must be consistent with $L_{j}$. Since $L_{j}=L_{k}$, we know that $\left(b_{j,k},ID_{j,k}\right)$ is consistent with $L_{k}$. □

Thus, any receiver $P_{k}$ can conclusively deduce about any other receiver $P_{j}$ what follows:

- If $P_{j}$ has sent a message consistent with $L_{k}$, then $P_{j}$ is honest.
- If $P_{j}$ has sent a message inconsistent with $L_{k}$, then $P_{j}$ is dishonest.
- If $P_{j}$ has sent ⊥, then $P_{j}$ may be honest or dishonest. However, if in this case $P_{j}$ is honest, then $P_{1}$ must be dishonest.

The rationale of Criterion (a) follows from Theorem 1. $P_{k}$ can conclude that $P_{i}$ and $P_{j}$ are honest when $\left(b_{i,k},ID_{i,k}\right)$ and $\left(b_{j,k},ID_{j,k}\right)$ are consistent with $L_{k}$. Now, if in addition $b_{i,k}\neq b_{j,k}$, $P_{k}$ can safely conclude that the sender ($P_{1}$) is dishonest. Consequently all the messages are not trustworthy and the output ⊥ is adequate for the situation.

As for Criterion (b), according to Theorem 1, we may conclude that all the receivers from the set *H* are honest and all others are not. Thus, *H* is the set of all honest receivers and their common message is trustworthy. Criterion (c) is similar to Criterion (b). Receivers from *H* here are also honest. However, in this case, some participants who are not in *H* may also be honest. The honest ones finally will change their output value from ⊥ to $b_{j,k}$. For safety reasons with respect to the agreement condition of DBA presented in Definition 2 by Criterion (d) in all other cases honest parties abort our protocol by setting their output to ⊥.

## 3. Analysis of the Protocol

Now, let us analyze the performance of our protocol under an attack of an adversary. We make the following assumption about the adversary:

1. The adversary can control a fixed set of participants and let those participants send arbitrary messages at his will. A participant is dishonest if and only if he is controlled by the adversary. The amount of honest participants is ≥3.
2. The adversary can bribe the list distributors to disclose certain information. When being bribed, a list distributor will disclose information with probability *p*.
3. The adversary has unlimited computing power.

In short, the adversary is static, Byzantine and with unlimited computing power.

**Theorem** **3.** *Our protocol satisfies agreement and validity under the attack of an adversary.*

**Proof.**  It is easy to see that validity is satisfied. Indeed, if none of the participants is controlled by the adversary, then they behave as the protocol specifies. Even if the adversary collects information from a large number of list distributors, the correlated list of numbers will still be correctly distributed. All participants will send consistent messages and the same output value will be established.

We now turn to the proof of agreement. First, note that the adversary can hardly have complete information of the combined reference lists $\left(L_{1},\ldots ,L_{n}\right)$. By our assumption, every list distributor is semi-honest. They will disclose the content of the list that they distributed with probability p<1, if the adversary bribes them. Since every list distributor contributes only $\frac{1}{d}$ to the lists, to collect complete information about $L_{1},\ldots ,L_{n}$, the adversary must bribe all *d* list distributors and still the probability of collecting complete information is $p^{d}$, which decreases exponentially as *d* grows. For those list distributors that the adversary does not bribe, the adversary cannot collect any information because the lists are distributed by quantum secure communication. The unlimited computing power the adversary has is not helpful in this case. Therefore, we conclude that the first stage of our protocol can be correctly and safely executed.

Now, we consider the second and third rounds. If the sender is honest, then there are at least two honest receivers. All honest receivers will receive the same consistent data from the sender. Those honest receivers will forward the same data to other participants. Therefore, according to Criterion (b) in our protocol, all honest participants will output the same value as the sender. If the sender $P_{1}$ is dishonest, then there are two cases:

1. All honest receivers receive consistent data. In this case, there are two sub-cases:
  (a) All honest receivers receive the same data. In this case, according to Criterion (b), all honest participants will output the same value.
  (b) Not all honest receivers receive the same data. Then, according to Criterion (a), all honest receivers will abort the protocol (output ⊥).
2. Not all honest receivers receive consistent data. In this case, if there are still two receivers that receive the same and consistent data and all other receivers output ⊥, then, according to Criterion (c), all honest receivers will output the same value. Otherwise, according to Criterion (a) or Criterion (d), all honest receivers will output ⊥.

Therefore, in all possible cases, the agreement is achieved. □

The above proof also implies an interesting property of our protocol which is stronger than validity. We present it as a corollary.

**Corollary** **1.** *Our protocol satisfies the following honest-success property under the attack of an adversary: if the sender is honest, then all honest parties decide on the same output as the sender.*

## 4. Conclusions and Future Work

We propose a protocol of quantum communication to achieve detectable Byzantine agreement among multiple parties. The significant feature of our protocol, compared to most existing protocols, is that it does not use entanglement. The success of our protocol relies on the distribution of sequences of reference lists, which in turn relies on the unconditional security of QKD. The way QKD is obtained is beyond the scope of the paper; we can just mention that in principle QKD can also be implemented without entanglement even if in some proposals the performance of QKD is improved by using two-qubit entangled state [16].

We also assume the participation of semi-honest list distributors in the protocol. This assumption is the price to pay for not using entanglement. Since low-dimensional entanglement can be implemented by current technology, in the future, we will study whether semi-honest distributors could be replaced by low-dimensional entanglement. One potential application of our DBA protocol is in the field of quantum blockchain [21,22,23]. In the future, we plan to apply our protocol to quantum blockchain to solve particular problems such as auction, lottery and multi-party secure computation.
